# *Akkermansia muciniphila*- and Pathogenic Bacteria-Derived Endotoxins Differently Regulate Human Dendritic Cell Generation and γδ T Lymphocyte Activation

**DOI:** 10.3390/biom14121571

**Published:** 2024-12-09

**Authors:** Veronica Fertitta, Barbara Varano, Manuela Del Cornò, Paola Fortini, Anna Aureli, Lucia Conti

**Affiliations:** 1 Department of Environment and Health, Istituto Superiore di Sanità, 00161 Rome, Italy; veronica.fertitta@iss.it (V.F.); paola.fortini@iss.it (P.F.); 2 Center for Gender-Specific Medicine, Istituto Superiore di Sanità, 00161 Rome, Italy; barbara.varano@iss.it (B.V.); manuela.delcorno@iss.it (M.D.C.); 3 Institute of Translational Pharmacology, National Research Council, 67100 L’Aquila, Italy; anna.aureli@cnr.it

**Keywords:** inflammation, innate immunity cells, microbiota, *Akkermansia muciniphila*, dendritic cells, lipopolysaccharide, lymphocytes

## Abstract

Lipopolysaccharide (LPS) is a potent endotoxin released at high concentrations in acute infections, causing massive host inflammatory response. Accumulating evidence indicates that dysbiosis-associated chronic low levels of circulating LPS can sustain a prolonged sterile low-grade inflammation that increases the risk of several non-communicable diseases. Interventions aimed at increasing the abundance of beneficial/probiotic bacteria, including *Akkermansia muciniphila*, result in reduced inflammation, favoring metabolic and immune health. Immunosuppression is a common feature in conditions of chronic inflammation, and dendritic cells (DCs) represent key targets given their ability to shift the balance toward immunity or tolerance. In this study, the effects of low concentrations of LPS from pathogenic (*Escherichia coli* and *Salmonella enterica*) and probiotic (*Akkermansia muciniphila*) bacterial species on human DC generation and functions were compared. We report that monocyte precursor priming with *Escherichia coli* and *Salmonella enterica* LPS forces the differentiation of PD-L1-expressing DCs, releasing high levels of IL-6 and IL-10, and impairs their capacity to drive full TCR-Vδ2 T cell activation. Conversely, comparable concentrations of *Akkermansia muciniphila* promoted the generation of DCs with preserved activating potential and immunostimulatory properties. These results shed light on potential mechanisms underlying the impact of low endotoxemia on disease risk and pathogenesis, and increase our understanding of the immunomodulatory effects of *Akkermansia muciniphila*.

## 1. Introduction

The mammalian gut harbors a wide variety of bacteria, thegastrointestinal (GI) microbiota, that coexist in a mutually beneficial state with their host and contribute to maintaining homeostasis. Accumulating evidence supports the role of intestinal dysbiosis in the onset and progression of GI-tract-associated disorders and several diseases involving distal organs and tissues [1]. Altered gut microbiota composition, mostly induced by unhealthy diets and lifestyles, and increased gut barrier permeability or leaky gut act as drivers for luminal bacteria and bacterial endotoxins to both colonize intestinal mucosa and mesenteric lymph nodes, and enter the bloodstream, with subsequent endotoxemia and chronic subclinical systemic inflammation. Lipopolysaccharide (LPS), the main component of the outer membrane of Gram-negative bacteria, is known to be a potent endotoxin released during acute bacterial infections that causes, through its lipid A component, a TLR4-mediated massive host inflammatory response that can degenerate into severe sepsis [2]. However, recent data suggest that persistent/chronic low levels of circulating microbiota-derived LPS can sustain a prolonged sterile low-grade inflammation, that increases the risk of several non-communicable diseases (NCDs) such as metabolic and cardiovascular diseases as well as neurodegenerative disorders and cancer [3,4,5]. It is speculated that LPS and other microbial molecular patterns are released in the luminal compartment during bacterial lysis, dissociated or escaped from the microorganism during growth, or included in outer membrane vesicles released by live bacteria [6,7,8,9]. Recently, LPS-positive extracellular vesicles, which were found to increase in the blood of obese mice, have been suggested as the mediators of the communications between the gut microbiota and extra-intestinal tissues [10]. Detectable LPS concentrations, albeit much lower (up to 50 times) than those found during acute infections, have been measured in the blood of subjects with obesity, diabetes, neurodegenerative disorders, and cancers and in the tumor tissue of colorectal cancer (CRC) patients [11,12,13,14,15,16]. In this regard, selective LPS blocking inside CRC tissue was shown to attenuate metastatic cancer progression and boost PD-L1-targeting immunotherapy [17,18]. Interestingly, LPS can be detected in the circulation of healthy individuals without causing any inflammatory reaction, with levels transiently increasing following consumption of fat-enriched foods [19,20].

The proinflammatory potential, or endotoxicity, of lipid A within the LPS molecule varies among Gram-negative bacterial species according to its structure and acylation/phosphorylation degree, that influence receptor recognition [4]. LPS molecules from some bacterial species have been reported to exert lower or even opposite effects on host immune/inflammatory response as compared to *Escherichia coli* (*E. coli)*- or *Salmonella enterica* (*S. enterica*)-derived endotoxins [21,22], and the relative abundance of microbial species may strongly affect the final host response by altering their balance.

Dysbiosis is characterized by a loss of bacterial diversity and altered microbial composition and often manifests as a contraction of the highly abundant obligate anaerobic bacteria belonging to the *Bacillota* and *Bacteroidota phyla* and a corresponding outgrowth of *Pseudomonadota*. Among these, expansion and acquired pathogenicity have been reported for members of the family Enterobacteriaceae, including, along with many harmless symbionts, several Gram-negative pathogens belonging to the genera Klebsiella, Shigella, Yersinia, Salmonella, and Escherichia [23]. Enriched gut commensal *E. coli* isolates were found to acquire the capacity to drive or exacerbate intestinal inflammation in recipient mice, largely through the induction of IL-6 [24].

With respect to the homeostatic condition, an increased Pseudomonadota/Verrucomicrobiota ratio has been reported in chronic intestinal inflammation and metabolic diseases [25]. Within the Verrucomicrobiota phylum, *Akkermansia muciniphila* (*A. muciniphila*) has been attracting great interest in recent years for its documented role in gut health. *A. muciniphila* is a commensal Gram-negative obligatory anaerobe that causes mucus degradation and produces SCFA, contributing to gut barrier integrity, metabolic health, and immune homeostasis [26]. Moreover, this microorganism has been involved in limiting LPS-induced inflammation in both the intestinal mucosa and the kidneys [27,28].

Interventions aimed at increasing *A. muciniphila* levels have reduced inflammation and enhanced gut health, with positive effects on the onset and progression of many chronic NCDs, such as metabolic syndrome, obesity, diabetes, IBD, and neurodegenerative diseases [28]. Furthermore, the baseline abundance of this microorganism has been associated with reduced colon tumorigenesis and the increased therapeutic potential of both conventional chemotherapy and immune checkpoint inhibitors, mostly through the activation of specific Th1 responses and suppression of Th17 polarization [26,29]. Nowadays, *A. muciniphila* is studied as a potential therapeutic target and a promising next-generation probiotic, and the pasteurized microorganism has been recently approved as a novel food by EFSA (Safety of Pasteurised Akkermansia muciniphila as a Novel Food Pursuant to Regulation (EU) 2015/2283|EFSA. Available online: https://www.efsa.europa.eu/en/efsajournal/pub/6780. Accessed on 15 October 2024). Several studies have indicated that the outer membrane compounds of *A. muciniphila* (postbiotics), or pasteurized bacteria, have greater therapeutic potential than live bacteria, although the exact mechanisms of action have not been fully understood [25]. Moreover, the effectiveness and safety of their supplementation in humans need further validation.

Microbiota–immune system interactions are highly complex and often confer mutual benefits to the host and resident microbes [30,31]. In the gut, immune cells are in close contact with luminal bacteria and contribute to immune homeostasis by preventing aberrant immune responses to harmless antigens and microbes and promoting host defense against pathogens. However, increased epithelium permeability and subsequent bacteria and bacterial product translocation toward mucosal tissue and into the bloodstream break this symbiotic alliance, leading to immunological dysregulation. Dendritic cells (DCs) are the most potent professional antigen-presenting cells acting as sentinels in early innate response and as a bridge between innate and adaptive immunity by initiating and guiding T cell responses [32]. In the gut, DCs sample foreign antigens and luminal microorganisms and contribute to maintaining intestinal immune homeostasis. Host systemic signals as well as foreign and endogenous factors within the tissue microenvironment can influence DC differentiation and switch their functions toward immunity or tolerance [32]. The mechanisms of DC dysfunction in cancer and chronic diseases are multiple. They are often related to their immature or semi-mature state, expression of inhibitory molecules such as PD-L1, release of inflammatory/immunosuppressive mediators, and STAT3 hyperactivation, all disrupting their proper communication with T cells and other immune cell populations [33,34].

γδ T lymphocytes, particularly the Vδ2 subset, are relevant players in the natural host defense against infections and malignancies; they are potent effectors of the antitumor response, as a major innate source of IFN-γ, and function through direct, MHC-unrestricted cytotoxic activity [35]. These characteristics have made them attractive candidates for alternative cancer immunotherapy strategies, and amino-bisphosphonate drugs, such as zoledronic acid, are being exploited to trigger their ex vivo or in vivo expansion [35]. A functional cross-talk of potential clinical interest between amino-bisphosphonate-exposed DCs and γδ T cells has been previously reported. Several pieces of evidence support the interplay between DCs and γδ T lymphocytes as a necessary critical process to generate an integrated immune response against cancer; thus, combined therapeutic strategies are under investigation [36,37]. However, within the immunosuppressive tumor microenvironment (TME), recruited Vδ2 T cells can be reprogrammed, switching their function toward cancer promotion [38]. Reduced Vδ2 T cell frequency and IFN-γ production have been reported in some inflammation-based pathologies, such as obesity and CRC [39,40]. The driving factors and mechanism(s) contributing to DC and γδ T cell contraction and to their improper polarization in cancer and inflammation-driven diseases have been only partially clarified.

Virulence factors released following acute bacterial infections and in the setting of sepsis, particularly LPS, have been extensively reported to target immune cells and manipulate host local and systemic immune responses to favor microorganism growth [41]. In particular, acute *E. coli* and *S. enterica* infection, as well as in vitro treatment with the corresponding LPS, was shown to completely inhibit DC differentiation in both mice and humans and consequently impair conventional T lymphocyte activation [42,43,44,45,46]. Conversely, whether LPS can affect the activation and expansion of γδ T lymphocytes has not been explored yet.

Although much of the research into the immunomodulatory effects of LPS has been carried out in the setting of infectious inflammation, LPS signaling is equally relevant to the pathophysiology of many chronic non-communicable diseases (NCDs). Nevertheless, only a few studies have attempted to dissect the immunological alterations induced by low LPS concentrations and to compare the effects on immune responses of endotoxins from pathogenic and beneficial commensal bacterial species. Regarding *A. muciniphila*-derived LPS, except a recent study showing modulatory effects on mouse microglia, no evidence has been reported yet on its possible regulatory role in human immune responses [47].

In this study, we investigated the effects of human monocyte precursor conditioning with low subclinical doses of *E. coli*- and *S. enterica*-derived LPS on in vitro generation of DCs and on their functional crosstalk with γδ T lymphocytes. In addition, the impact of comparable doses of LPS from *A. muciniphila* was studied to deeply understand the immunomodulatory capacity of this commensal microorganism.

## 2. Materials and Methods

### 2.1. Ethics Statement

Buffy coats from healthy donors were obtained from Centro Trasfusionale, Sapienza University of Rome; they were not obtained specifically for this project. Their use in this study was approved by the Institutional Review Board of Istituto Superiore di Sanità (AOO-ISS—22 March 2022—0010603 Class: PRE BIO CE 01.00). According to the Italian law n. 219 of 21 October 2005, revised 2 November 2015 (D.L. of the Ministry of Health), donors signed an informed consent. Centro Trasfusionale treated all data according to the Italian law on personal data management “Codice in materia di protezione dei dati personali” (Testo unico D.L. 30 June 2003 n. 196).

### 2.2. Dendritic Cell Generation and Culture

Monocytes were isolated from the peripheral blood of healthy donors (25 total donors, mean age 46 ± 14, male/female 2:1). Peripheral blood mononuclear cells (PBMCs) were separated by Ficoll/Paque density gradient centrifugation and monocytes were then isolated by immunomagnetic selection using CD14+ microbeads (MACS monocyte isolation kit, Miltenyi Biotec, Auburn, CA, USA). This procedure yields a 95% pure monocyte population as assessed by analysis of lineage-specific markers. Soon after seeding, monocytes (1 × 10^6^ cells/mL) were left untreated or exposed to different concentrations of LPS (phenol extraction purified, 0.01 to 10 ng/mL) derived from *E. coli* (EH100 serotype, ≥5 × 10^5^ EU/mg, Alexis Biochemicals Corp., Nottingham, United Kingdom), *S. enterica* (S. minnesota R345 serotype, ≥5 × 10^5^ EU/mg, Alexis Biochemicals Corp.), and *A. muciniphila* (≥1 × 10^4^ EU/mg, Sigma-Aldrich, St. Louis, MO, USA). Dendritic cells (DCs) were generated by culturing unprimed and LPS-primed monocytes for 5 days in RPMI 1640 medium (endotoxin content < 0.005 EU/mL, EuroClone, Pero, Italy) supplemented with 10% heat-inactivated fetal bovine serum (final endotoxin content < 0.008 EU/mL), in the presence of recombinant granulocyte–macrophage colony-stimulating factor (GM-CSF, 50 ng/mL, kindly provided by Schering-Plough, Dardilly, France) and IL-4 (500 U/mL, Miltenyi Biotec, Auburn, CA, USA). Fresh medium plus cytokines were added after 3 days of culture. In selected experiments, monocytes were simultaneously treated with *A. muciniphila* and *E. coli* LPS or pre-treated with *A. muciniphila* LPS for 0.5, 2, and 4 h prior to *E. coli* LPS exposure. To induce DC maturation, immature cells were stimulated for the last 24 h of culture with 10 ng/mL of *E. coli* LPS. In some experiments, DCs were induced to maturstion, following exposure to *S. enterica or A. muciniphila* LPS (10 ng/mL), or the TLR8 agonist R848 (1 µg/mL, InvivoGen, San Diego, CA, USA).

### 2.3. Detection of Cell Viability via Trypan Blue Dye Staining

The percentages of live monocyte-derived DC was assessed on day 5 of culture by Trypan blue exclusion test of cell viability. Briefly, cells were harvested, mixed with the dye (1:1 dilution), and counted on the TC20 automated Cell Counter (Biorad, Hercules, CA, USA).

### 2.4. γδ T Lymphocyte Isolation and DC/γδ T Cell Cocultures

PBMCs and the eluted fraction of CD14 negative cells (peripheral blood lymphocytes, PBL) were cryopreserved for subsequent γδ T cell isolation and coculture experiments. γδ T lymphocytes were isolated from PBMC by positive selection using the anti-TCRγ/δ MicroBead Kit (Miltenyi Biotec, Auburn, CA, USA) and cultured overnight in complete medium (RPMI plus 10% FBS) at 37 °C to allow bead release. After extensive washing, purified γδ T lymphocytes were cocultured with unprimed and LPS-primed autologous DC (ratio 1:1), in the absence or presence of zoledronate (ZOL, 5 µg/mL, kindly provided by Novartis Pharma, Basel, Switzerland) for 48 h, as previously described [48]. Parallel cocultures of DC and autologous total PBL (ratio 1:1) were set up, stimulated with ZOL (5 µg/mL), and incubated for 6 days at 37 °C to allow Vδ2 T cell expansion.

### 2.5. Phenotypic Analysis of DC and γδ T Lymphocytes

Five-day-cultured immature and mature DCs were analyzed for the expression of surface markers by staining with phycoerythrin (PE)-conjugated mAbs to CD1a, CD80, MHC class II, CD206, CD274, CD83, and CD86, respectively, or with isotype-matched control mAbs (BD Biosciences, San Diego, CA, USA). Briefly, 10^5^ cells were preincubated with phosphate-buffered saline containing 10% human AB serum to block unspecific Ig binding, then stained with the specific mAbs for 30 min on ice, washed, and analyzed by flow cytometry. γδ T cells were stained with PE-conjugated mAbs to CD25 and MHC class II (BD Biosciences, San Diego, CA, USA) and fluorescein isothiocyanate (FITC)-conjugated mAb to TCR-Vδ2 (Miltenyi Biotec, Auburn, CA, USA) or with isotype-matched control mAbs for 30 min on ice, washed, and analyzed by flow cytometry. At least 10,000 events/sample were acquired by a FACScan cytometer (BD Biosciences, San Diego, CA, USA). Data analysis was performed by gating on the lymphocyte population and excluding dead cells and debris.

### 2.6. Antigen Uptake and Processing

DC cultures were pulsed with the self-quenched DQ Ovalbumin (DQ-OVA, 1 mg/mL, Molecular Probes) for 15 min at 37 °C. Cells were then extensively washed and incubated at 37 °C or 0 °C (as control) for 30 min. Processing of OVA protein into peptides was measured by flow cytometry as an increase in fluorescence intensity over time. At least 10,000 events/sample were acquired by a FACScan cytometer (BD Biosciences, San Diego, CA, USA).

### 2.7. Cytokine Release Determination

Culture media, collected from 5-day immature and mature DC cultures, and from 48 h DC/γδ T cell cocultures, were analyzed by enzyme-linked immunosorbent assay (ELISA) for the release of IL-1β, IL-6, IL-10, IL-12, IFN-γ (Biolegend. San Diego, CA, USA), and IL-17 (R&D Systems Inc., Minneapolis, MN, USA), respectively.

### 2.8. Immunoblotting Analysis

Protein extracts were obtained from 5-day-cultured DCs by lysing cells with RIPA buffer [150 mM NaCl, 50 mM Tris-Cl (pH 7.5), 1% Nonidet P-40, 0.5% sodium deoxycholate, and 0.1% sodium dodecyl sulfate (SDS)] plus protease and phosphatase inhibitors (Sigma-Aldrich, St. Louis, MO, USA). Proteins were fractionated on NuPAGE 4–12% Bis-Tris Gel (Thermo Fisher Scientific, Waltham, MA, USA), transferred to a nitrocellulose membrane and subjected to immunoblot analysis using mouse or rabbit antibodies specific for either the total or the tyrosine phosphorylated forms of STAT3 and for PD-L1 (Cell Signaling Technology, Beverly, MA, USA). Equal loading of proteins was verified by blotting the same gel with antibodies to the housekeeping gene GAPDH (Santa Cruz Biotechnology, Santa Cruz, CA, USA).

### 2.9. Statistical Analysis

GraphPad Prism 8 software was used for statistical analysis. Statistical comparisons were performed using the two-tailed paired Student’s *t*-test for independent samples. A *p* value ≤ 0.05 was considered statistically significant.

## 3. Results

### 3.1. Priming of Human Monocyte Precursors with Low Dose LPS from E. coli and S. enterica, but Not from A. muciniphila, Leads to the Generation of PD-L1-Expressing Dendritic Cells with Preserved Functions

**Figure 1:**
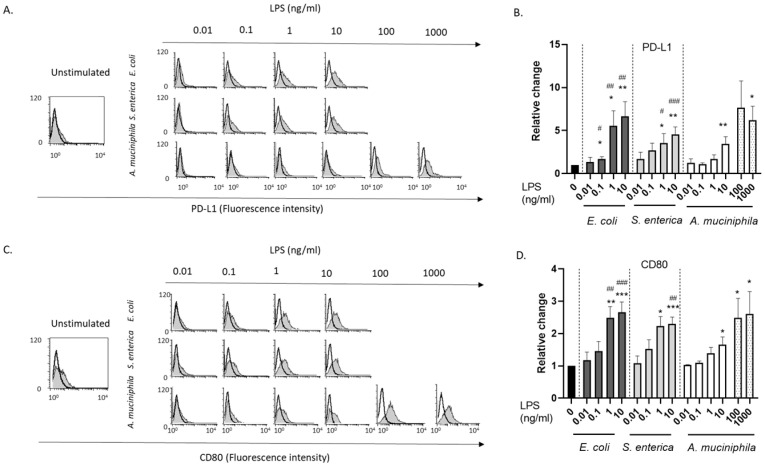
Surface PD-L1 and CD80 expression in human DC cultures conditioned with increasing doses of LPS. Human peripheral blood monocytes were induced to differentiate toward DCs in standard medium in the absence or presence of the indicated concentrations of LPS from *E. coli*, *S. enterica,* and *A. muciniphila*. Cells were analyzed on day 5 of culture by flow cytometry for the expression of surface PD-L1 (**A**,**B**) and CD80 (**C**,**D**). Results from one representative donor are shown in panels A and C. The dark-open histograms represent background staining of isotype-matched control Ab, the light gray-filled histograms represent cells stained with the indicated Ab. The relative change of the percentages of PD-L1- and CD80-positive cells in LPS-primed versus unprimed cultures is reported in (**B**–**D**). The unprimed control cultures (LPS = 0 ng/ml) are set as 1.0. Mean values  ±  SE from 15 and 10 independent donors are shown in B and D, respectively. Statistical significance was indicated versus untreated control (*) or comparable concentrations of *A. muciniphila* LPS (#). */# 0.01 < *p*  ≤  0.05; **/## 0.001 < *p*  ≤  0.01; ***/### *p*  ≤  0.001.

We tested the hypothesis that endotoxins from pathogenic and beneficial bacteria, at concentrations mimicking those achieved in conditions of dysbiosis or transient increase in epithelium permeability, can differently affect DC generation and function. To this aim, human peripheral blood monocytes isolated from healthy donors were exposed to LPS (0.01 to 10 ng/mL) from *E. coli*, *S. enterica,* or *A. muciniphila*, and allowed to differentiate toward DCs; then, phenotypic and functional analyses were performed. All unstimulated and LPS-stimulated cultures exhibited dendritic cell morphology; however, the appearance of some spindle-shaped cells as well as a lower viability was repeatedly reported in *E. coli* and *S. enterica* LPS-stimulated cultures as compared to those exposed to comparable concentrations of *A. muciniphila* LPS (Figure S1). A significant increase in the percentage of PD-L1-expressing cells was observed in DC cultures generated in the presence of *E. coli*- and *S. enterica*-derived LPS, at concentrations as low as 0.1 ng/mL, compared to unstimulated cultures (Figure 1A,B). In contrast, a significantly lower proportion of PD-L1-positive cells was found in cultures exposed to the higher dose (10 ng/mL) of LPS from *A. muciniphila,* and no effect was reported at lower concentrations (Figure 1A,B). Similarly, an increase in the proportion of CD80-positive cells was achieved following monocyte priming with *E. coli* and *S. enterica* LPS, whereas a lower effect was elicited by *A. muciniphila*-derived endotoxin (Figure 1C,D). However, by increasing *A. muciniphila* LPS concentration up to 100–1000 ng/mL, the expression of both PD-L1 and CD80 was upregulated at comparable levels as with *E. coli* and *S. enterica* LPS at 10 ng/mL (Figure 1A–D). Moreover, a lower cell viability was observed in DC cultures exposed to high *A. muciniphila* LPS doses (Figure S1).

To highlight possible synergic or antagonistic effects of commensal and pathogenic-bacteria-derived endotoxins, monocytes were simultaneously exposed to *A. muciniphila* and *E. coli* LPS or treated with *A. muciniphila* LPS for 0.5, 2, or 4 h before priming with *E. coli* LPS, respectively. The expression of PD-L1 and CD80 in generated DC was not affected by these treatments (Figure S2), indicating that *A. muciniphila* endotoxin neither potentiates nor antagonizes the effects of *E. coli* LPS on DC phenotype.

Sex dimorphism in soluble PD-L1 release induced by LPS and inflammatory cytokines has been described in endothelial cells [49]. To assess whether sex-related differences in surface PD-L1 basal expression or in its regulation by LPS could be observed, we compared DC cultures generated from male and female donors. Baseline and LPS-induced levels of surface PD-L1 were not significantly different in DC cultures obtained from male and female donors (Figure S3).

**Figure 2:**
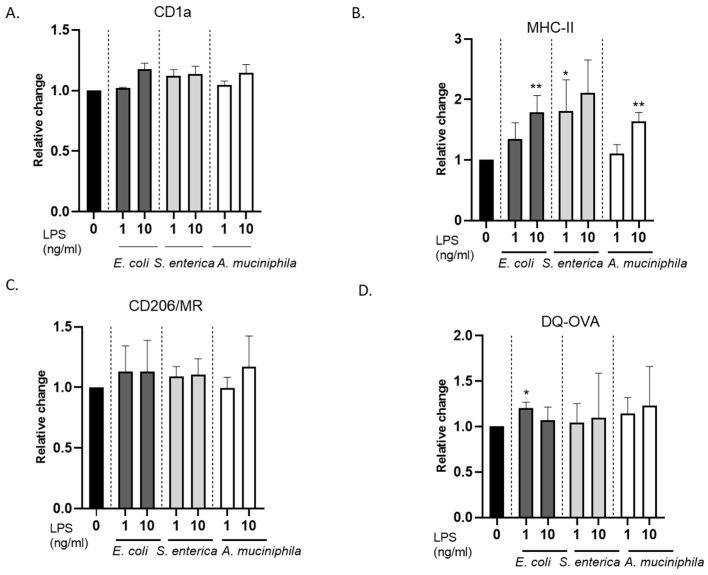
Phenotypic and antigen processing analyses of LPS-conditioned *DCs*. DC cultures were generated as described in the legend in Figure 1 and analyzed for the surface expression of CD1a (**A**), MHC class II (**B**), and CD206/MR (**C**) or ovalbumin processing (**D**) by flow cytometry. Data are shown as relative change of the median fluorescence intensity (MFI) in LPS-primed versus unprimed cultures and represent mean values  ±  SE of four independent donors. * 0.01 < *p*  ≤  0.05; ** 0.001 < *p*  ≤  0.01.

It has been reported that monocyte exposure to high, infection-mimicking *E. coli* LPS concentrations (0.1–1 µg/mL) results in impaired DC differentiation and reduced MHC expression and endocytic activity [46,47,49]. To check whether the 100-fold-lower LPS concentrations we used (1–10 ng/mL) could elicit the same effect, LPS-primed cultures were also analyzed for the expression of CD1a, MHC class II, and CD206/mannose receptors. Exposure of monocytes to 1–10 ng/mL of LPS from the different bacterial strains did not significantly interfere with CD1a induction and did not modulate CD206 expression (Figure 2A,B and Figure S4). In preliminary experiments, the parallel downregulation of CD14 was also verified. Conversely, the expression of MHC class II was found to increase in LPS-exposed as compared to untreated DC cultures, independently of the LPS source (Figure 2C and Figure S4). Moreover, consistent with the expression of CD206, ovalbumin uptake and processing occurred at comparable levels in all LPS-primed and unprimed cultures (Figure 2D and Figure S4).

Altogether, these findings indicate that low concentrations of LPS from pathogenic *E. coli* and *S. enterica* favor the generation of CD1a^+^ DC with preserved antigen uptake and processing capacity but potentially delivering inhibitory signals. Conversely, no inhibitory molecule induction was elicited by comparable doses of endotoxin from commensal/probiotic *A. muciniphila*.

### 3.2. A. muciniphila LPS-Conditioned DC Cultures Release Lower Amounts of Immune Mediators as Compared to E. coli and S. enterica LPS-Exposed Cultures

**Figure 3:**
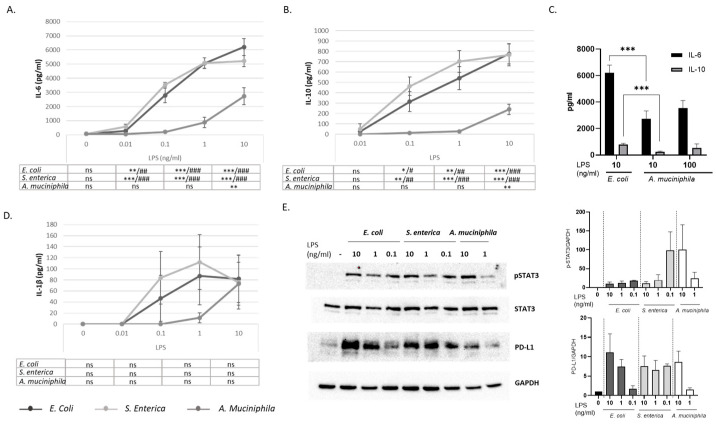
Effects of monocyte priming with LPS from different sources on cytokine production and STAT3 activation in differentiated DC cultures. DC cultures were generated in the absence or presence of the indicated concentrations of LPS, as described in the legend to Figure 1. Conditioned media were collected and analyzed for the secretion of IL-6 (**A**–**C**), IL-10 (**B**,**C**), and IL-1β (**D**) by ELISA. Data are expressed as mean values ± SE from twelve (**A**), eight (**B**,**D**), and (**C**) independent donors. Statistical significance was indicated versus untreated control (*) or comparable concentrations of *A. muciniphila* LPS (#). */# 0.01 < *p*  ≤  0.05; **/## 0.001 < *p*  ≤  0.01; ***/### *p*  ≤  0.001. (**E**) Western blotting analysis showing the levels of total STAT3, the tyrosine phosphorylated form pSTAT3 and PD-L1. (**E**) A representative blot is shown. Results from densitometric analysis were normalized to the housekeeping GAPDH content and are expressed as mean values ± SE of three independent experiments.

To investigate whether monocyte exposure to LPS from the different bacterial strains could differently affect the secretion of pro -and anti-inflammatory immune mediators in DC cultures, cell-conditioned media were analyzed for the release of IL-6, IL-10, and IL-1β. Figure 3A–D shows the levels of cytokine secretion following stimulation with *E. coli*, *S. enterica,* and *A. muciniphila* LPS at different concentrations. Significant dose-dependent induction of IL-6 and IL-10 release was observed in DC cultures generated in the presence of all LPS types compared to unstimulated cultures (Figure 3A,B). However, the levels of both cytokines were significantly lower in the cultures stimulated with *A. muciniphila* LPS compared to those exposed to *E. coli* and *S. enterica* endotoxins at all the concentrations tested (Figure 3A,B). Again, by increasing the concentration of *A. muciniphila* LPS up to 100 ng/mL, the difference with the other endotoxins in the induction of IL-6 and IL-10 was lost (Figure 3C). An induction of IL-1β secretion by all LPS types was also found; however, the high inter-donor variability did not allow us to highlight statistically significant differences among the different LPSs (Figure 3D).

IL-6 and IL-10 are both inducers of STAT3 activation. Furthermore, STAT3 has been involved in the induction of PD-L1 in TLR8-Stimulated monocytes [50]. We thus analyzed the expression of tyrosine-phosphorylated STAT3 in LPS-stimulated DC cultures. The results in Figure 3E show that, despite of reduced production of IL-10 and IL-6, the levels of phosphorylated STAT3 detected in *A. muciniphila* LPS-conditioned DCs were not significantly different from those found in *E. coli* and *S. enterica* LPS-primed cultures, suggesting that other molecular mechanism(s) might be involved in LPS-mediated PD-L1 induction. In line with this, the expression of total PD-L1 reflected the expression observed for the surface molecule (Figure 1A,B and Figure 3E).

### 3.3. DCs Conditioned by A. muciniphila but Not E. coli or S. enterica LPS Retain the Ability to Mature in Response to Subsequent TLR4 and TLR8 Triggering

**Figure 4:**
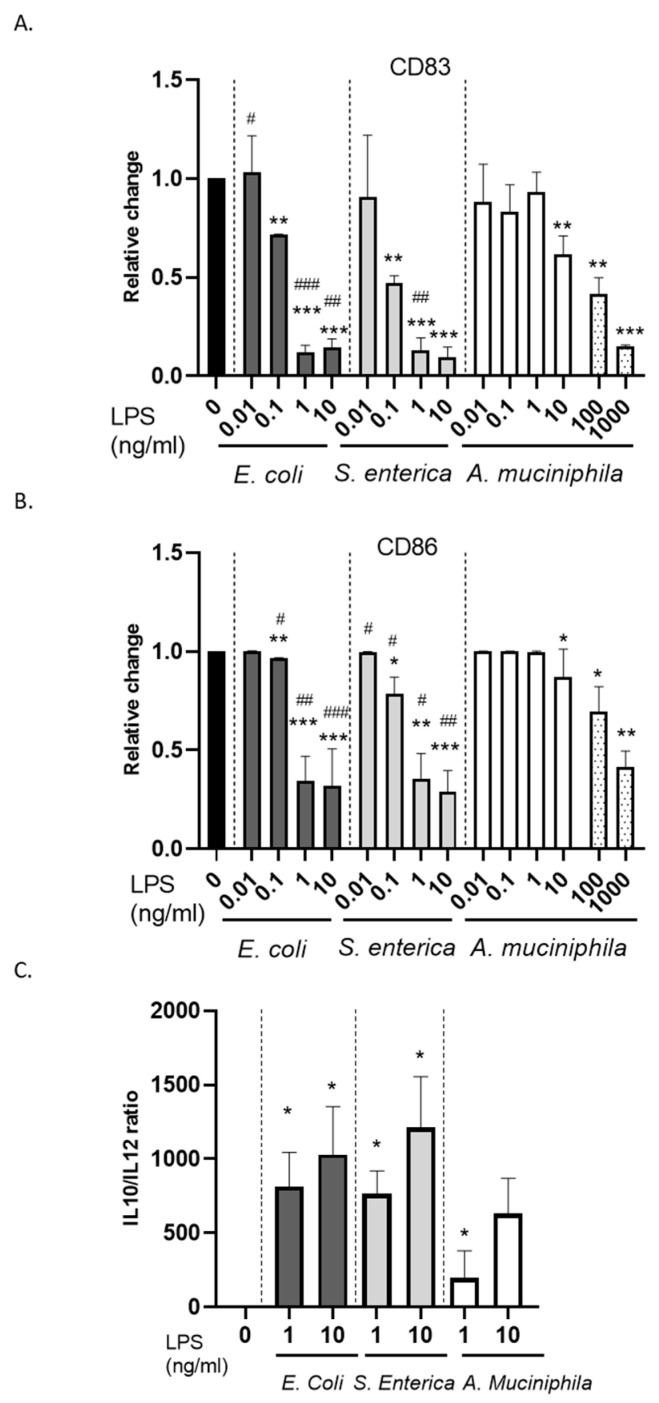
Effect of monocyte priming with LPS from the different bacterial strains on DC maturation. DCs were differentiated from monocytes exposed or not to increasing concentrations of the different LPSs and then left untreated or stimulated with *E. coli* LPS during the last 24 h of culture to induce maturation. (**A**,**B**) The expression of the surface activation markers CD83 and CD86 was analyzed by flow cytometry. Histograms indicate the relative change in the percentages of positive cells in LPS-primed versus unprimed cultures. Results are shown as mean values ± SE from ten independent donors. (**C**) Conditioned media were collected and analyzed for the secretion of IL-10 and IL-12. Results are shown as relative change in the IL-10/IL-12 ratio in LPS-primed versus unprimed DC cultures and are expressed as mean values ± SE from three to six independent donors, depending on LPS concentration. Statistical significance was indicated versus untreated control (*) or comparable concentrations of *A. muciniphila* LPS (#). */# 0.01 < *p*  ≤  0.05; **/## 0.001 < *p*  ≤  0.01; ***/### *p*  ≤  0.001.

We next analyzed the capacity of *A. muciniphila* LPS-conditioned DCs to activate in response to a subsequent LPS stimulation. Immature DC cultures generated in the absence or presence of endotoxins from the different bacterial strains were thus exposed, during the last 24 h of culture, to *E. coli* LPS and then analyzed for the expression of costimulatory molecules and cytokine release. It is well known that *E. coli* LPS exposure of monocytes induces tolerance mechanisms [51]. Accordingly, we reported that the induction of the activation markers CD83 and CD86 is strongly impaired in *E. coli*- and *S. enterica*-primed cultures at concentrations as low as 0.1 ng/mL (Figure 4A,B and Figure S5). In contrast, comparable doses of *A. muciniphila* LPS did not affect CD83 and CD86 upregulation (Figure 4A,B and Figure S5), thus failing to induce LPS tolerance. However, when *A. muciniphila* LPS dose was increased to 100 or 1000 ng/mL, its effect on DC maturation was not significantly different from that of *E. coli* and *S. enterica* endotoxins (Figure 4A,B and Figure S5). Consistent with the expression of maturation markers, *A. muciniphila* LPS-primed cultures exhibited a lower IL-10/IL-12 ratio compared to *E. coli*- and *S. enterica*-primed cultures, though higher with respect to control unstimulated cultures (Figure 4C). Furthermore, in contrast to *E. coli* and *S. enterica* endotoxins, *A. muciniphila* LPS failed to induce cross-tolerance towards the subsequent triggering of TLR8 (Figure S6).

### 3.4. Monocyte Priming with E. coli and S. enterica but Not A. muciniphila LPS Impair DC-Mediated Full γδ T Lymphocyte Activation

**Figure 5:**
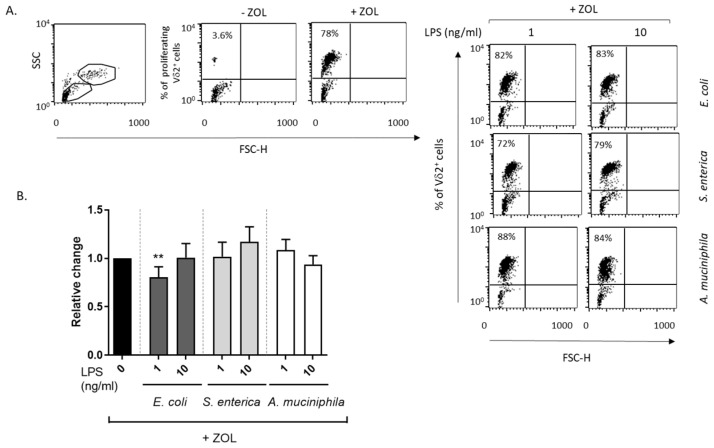
Effect of monocyte precursor priming with LPSs from the different bacterial strains on DC-mediated TCRVδ2 T cell expansion. DCs generated in the absence or presence of LPS from the indicated bacterial strains were cocultured with autologous PBL (1:1 ratio) and left unstimulated or stimulated with ZOL. Expansion of TCRVδ2 T cells within total PBL was analyzed after 6 days by flow cytometry gating on the lymphocyte population (R1), with each dot representing individual cell. (**A**) Results from one representative donor out of 16 analyzed are shown. R2, gated DC population. (**B**) The relative change of the percentages of TCRVδ2-positive cells in LPS-primed versus unprimed ZOL-stimulated cocultures is reported. Mean values  ±  SE from 16 independent donors are shown. Statistical significance was indicated versus untreated control (*). ** 0.001 < *p*  ≤  0.01.

We previously demonstrated that amino-bisphosphonate-stimulated DCs induce the activation and IL-2-Independent proliferation of γδ T lymphocytes [36]. To test the hypothesis that LPS conditioning of DCs impairs their functional interaction with γδ T lymphocytes, unprimed and LPS-primed DCs were cocultured with autologous PBL in the absence or presence of ZOL. Vδ2 T cell expansion was analyzed after 6 days by flow cytometry. Comparable proportions of Vδ2 T cells were found expanded in ZOL-stimulated control and LPS-conditioned cocultures as compared to unstimulated ones, independently of the LPS source, with only a slight reduction induced by *E. coli* LPS (Figure 5A,B). In a subgroup of donors, Vδ2 T cell staining was also performed at earlier time points (3 days) with similar results (Figure S7).

**Figure 6:**
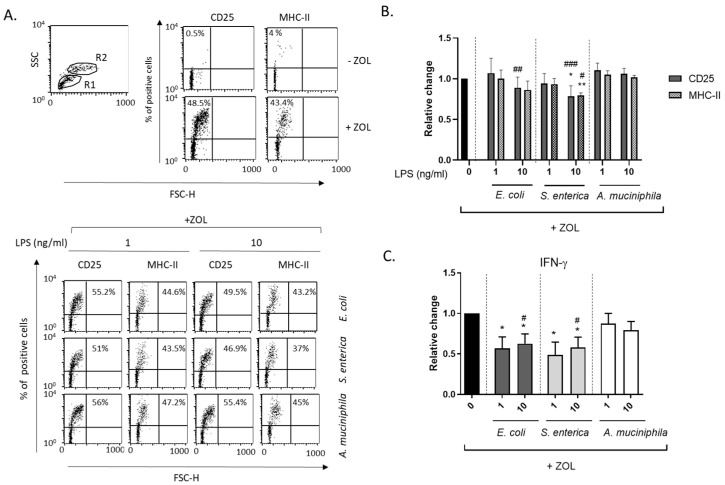
Effect of monocyte precursor priming with LPS from the different bacterial strains on DC-mediated *γδ* T cell *activation*. DCs generated in the absence or presence of LPS from the indicated bacterial strains were cocultured with purified autologous γδ T lymphocytes (1:1 ratio) and left unstimulated or stimulated with ZOL. (**A**,**B**) Expression of the activation markers CD25 and MHC class II by ZOL-stimulated γδ T cells was analyzed after 48 h by flow cytometry gating on the lymphocyte population (R1). R2, gated DC population. (**A**) Results from one representative donor out of five analyzed are shown. (**B**) The relative change of the percentages of CD25 and MHC class II positive γδ T cells in LPS-primed versus unprimed cocultures is reported. Mean values  ±  SE from 5 independent donors are shown. (**C**) Conditioned media from ZOL-stimulated DC/γδ T lymphocyte cocultures were analyzed for the secretion of IFN-γ. Results are reported as relative change of IFN-γ production (pg/mL) in LPS-primed versus unprimed cocultures and are expressed as mean values ± SE from five independent donors. (**B**,**C**) Statistical significance was indicated versus untreated control (*) or comparable concentrations of *A. muciniphila* LPS (#). */# 0.01 < *p*  ≤  0.05; **/## 0.001 < *p*  ≤  0.01; ### *p*  ≤  0.001.

To further investigate whether LPS conditioning could affect early Vδ2 T cell activation, purified total γδ T lymphocytes were cocultured with autologous unstimulated or LPS-exposed DCs, in the presence or absence of ZOL, and analyzed 48 h later for the induction of activation markers and cytokine expression. A low but significant reduction in the proportion of CD25- and MHC class II-expressing γδ T cells was found in cocultures with DCs conditioned by *S. enterica* LPS, whereas no effects were observed in cultures pre-exposed to *E. coli* or *A. muciniphila* LPS as compared to unprimed ones (Figure 6A,B). However, when cytokine release in the conditioned media was analyzed, a marked reduction in the secretion of IFN-γ was observed for ZOL-stimulated γδ T cells cocultured with both *E. coli* and *S. enterica* LPS-primed DCs (Figure 6C). Control experiments in which unstimulated DC/γδ T cell cocultures were exposed to *E. coli* and *S. enterica* LPS soon before the addition of ZOL did not result in IFN-γ reduction (1.13- and 0.97-fold change, respectively, relative to unstimulated cocultures, thus excluding any direct effect of residual endotoxins on lymphocytes). Conversely. *A. muciniphila* LPS-exposed DCs did not exert any inhibitory effect on IFN-γ production (Figure 6C), suggesting that pathogenic- and commensal/probiotic-bacteria-derived endotoxins differently impact γδ T cell functions.

Furthermore, based on recent data reporting a switch toward IL-17 production in a mouse model of LPS-induced inflammation, DC/γδ T cell cocultures were also analyzed for the release of IL-17. However, this cytokine was detected neither in LPS-conditioned nor in control cocultures.

### 3.5. A. muciniphila LPS Can Act by Itself as a DC Maturation Stimulus

**Figure 7:**
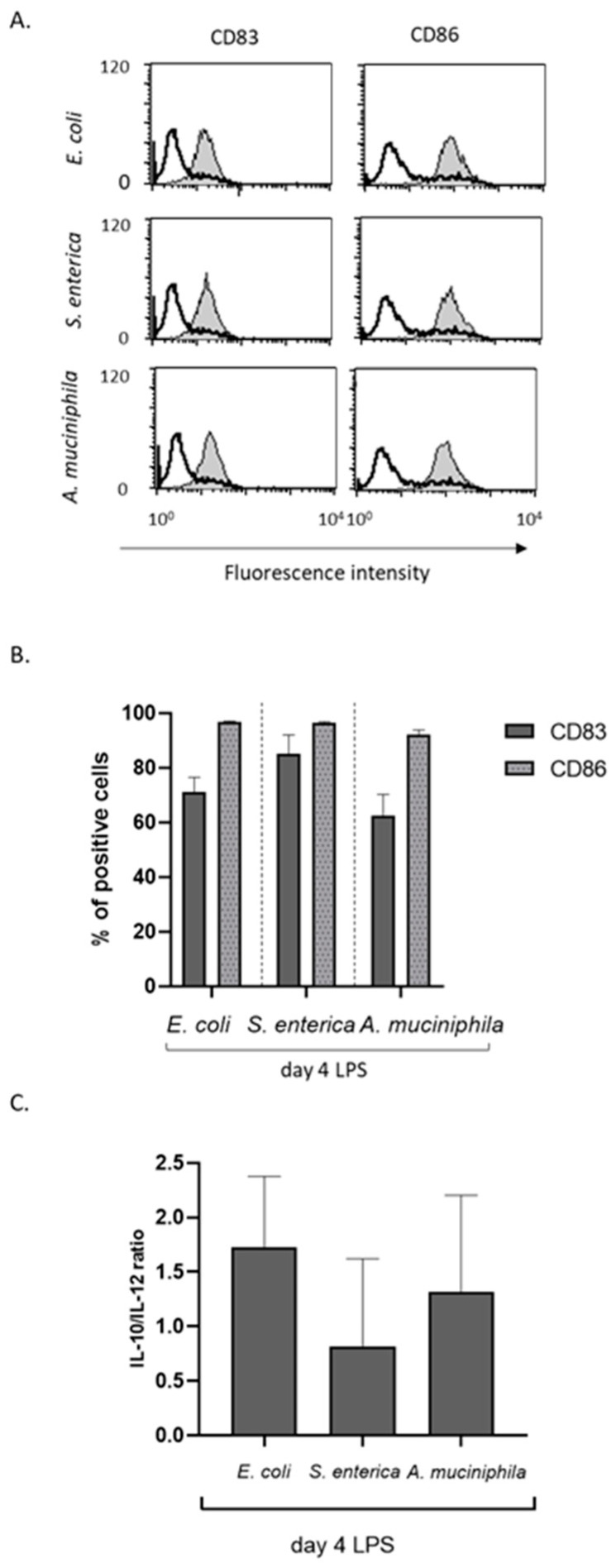
*A. muciniphila*-derived LPS induces the maturation of pre-existing immature DCs. DC cultures were generated from unprimed control monocytes and then exposed, during the last 24 h of culture, to LPS (10 ng/mL) from *E. coli*, *S. enterica,* and *A. muciniphila* to induce maturation. Cells were then analyzed for the expression of the activation markers CD83 and CD86 by flow cytometry (**A**,**B**) and the secretion of IL-10 and IL-12 by ELISA (**C**). (**A**) One representative donor out of the eleven analyzed is shown. The dark-open histograms represent the CD83 and CD86 staining of LPS unstimulated immature cultures, the light gray-filled histograms represent the CD83 and CD86 staining of LPS- stimulated cells. (**B**) The percentages of positive cells in cultures stimulated with LPS from the different sources are shown and results are represented as mean values ± SE from eleven independent donors. (**C**) Results are expressed as IL-10/IL-12 ratio and represented as mean values ± SE from three independent donors.

To investigate whether *A. muciniphila* endotoxin was as efficient as *E. coli* LPS in inducing the maturation of pre-existing immature DCs, DC cultures generated from unstimulated monocytes were exposed to 10 ng/mL of *E. coli*, *S. enterica,* or *A. muciniphila* LPS during the last 24 h of culture and then analyzed for activation marker and cytokine expression. As shown in Figure 7A,B, LPS from *A. muciniphila* upregulated CD83 and CD86 expression at comparable levels as *E. coli*- and *S. enterica*-derived endotoxins. Furthermore, a comparable IL-10/IL-12 ratio was measured in the conditioned media of all LPS-stimulated DCs, independently of the endotoxin source (Figure 7C), thus demonstrating that LPS derived from *A. muciniphila* acts as a DC maturation stimulus.

## 4. Discussion

Gut microbiota represents a substantial and dynamic source of endotoxins, which play important roles in immune modulation and can contribute to the development of chronic diseases [21]. Structural and functional diversity among LPS from different bacteria serotypes accounts for different degrees of antigenicity and immune toxicity; thus, the relative abundance of microbial species may deeply affect the final host response.

In this study, we compared the effects of low concentrations of LPS, derived from pathogenic or commensal/probiotic bacterial species, on the in vitro generation of DCs from human blood monocyte precursors and on their functional crosstalk with γδ T lymphocytes. Unlike the impairment of DC differentiation reported in previous studies using high concentrations of endotoxin [42,43,44,45,46], we demonstrate that pre-exposure of monocytes to *E. coli* and *S. enterica* LPS at concentrations as low as 0.1 ng/mL forces the generation of PD-L1-expressing CD1a^+^ DCs, which release high levels of IL-6 and IL-10. Furthermore, endotoxin conditioning was found to impair the capacity of DC to drive the full activation of γδ T lymphocytes and to respond to a subsequent encounter with either bacterial or viral molecular patterns. Based on these observations, we can speculate that, when dysbiosis occurs and pathogenic Gram-negative bacteria are predominant, released LPS, albeit at lower concentrations than those achieved in acute infections, can put a stiff challenge to immune cells and create an immunosuppressive environment. Conversely, it can be envisaged that, under healthy conditions, LPS derived from beneficial commensal bacteria would act to prime immune cells and keep the immune system battle-ready. In support to this hypothesis, our results show that monocyte priming with comparable concentrations of LPS from commensal *A. muciniphila* promotes the generation of DC with preserved immunostimulatory activity and fails to induce tolerance or cross-tolerance to subsequent TLR triggering.

The balance between more and less toxic LPS is influenced by the relative abundance of the various types of microorganisms and the final response may depend on a mixture of low- and high-endotoxicity LPSs. As the different LPSs can compete for the receptors on immune cells, changing their relative ratios might be used to reduce the deleterious effects on immunity and inflammation. In this regard, lifestyle, particularly dietary habits, deeply affect the relative microbial species abundance [52]. Furthermore, microorganisms with probiotic properties have been exploited for this purpose [53]. In recent years, *A. muciniphila* has been widely studied for its use as a potential probiotic, due to its capacity to maintain gut barrier integrity and to improve metabolic and immune health [26]. Although the underlying mechanism(s) have not been fully understood, several studies have indicated that the outer membrane compounds, or the pasteurized microorganisms, have greater preventive or therapeutic potential than live bacteria [25]. Our results show for the first time that human monocytes primed with *A. muciniphila* LPS retain their capacity to differentiate toward immunostimulatory DC exhibiting a low expression of PD-L1 and immunosuppressive cytokines. At the same time, we report that *A. muciniphila* LPS can induce, by itself, the activation of pre-existing immature DCs, thus further sustaining immune responses. Furthermore, *A. muciniphila* LPS conditioning of DCs does not interfere with their capacity to trigger γδ T lymphocyte activation, in contrast to comparable doses of pathogenic-bacteria-derived endotoxins. Altogether, these findings increase our understanding of the behavior of this microorganism and its membrane components, particularly of its interaction with human immune cells, and further support its use as a probiotic. In this regard, it has been recently demonstrated that DC treatment with whole *A. muciniphila* or its outer membrane vesicles leads to tolerogenic DCs, showing increased IL-10 and reduced IL-12 production [54]. Likewise, *A. muciniphila* promotes an anti-inflammatory phenotype in human macrophages [55]. Nevertheless, we report herein that increasing the concentrations of *A. muciniphila* LPS leads to equally suppressive effects on DC functions as with pathogenic bacteria derived endotoxins. Accordingly, previous studies have shown that intestinal colonization by this microorganism can exacerbate inflammation [56,57]. It is worth noting that LPS constitutes the bulk of the bacteria outer layer, with around 10^6^ molecules accounting for the three-quarters of the cell surface, suggesting that high concentrations can be reached within the intestinal lumen in particular conditions [3]. Furthermore, the heat-stable nature of endotoxins should be considered [58]. Although promising preclinical studies led to the approval of pasteurized *A. muciniphila* as a novel food by EFSA, the mechanism(s) of action of the entire microorganism as well as of its structural components need to be further and carefully explored, and the effectiveness and safety of its supplementation in humans further validated.

LPS from some bacterial species, such as *B. fragilis* and *B. dorei*, have been shown to elicit a lower response in human PBMC compared to the proinflammatory *E. coli* LPS, and also to directly antagonize the TLR4-mediated effects of the latter, likely depending on their uncanonical lipid A structure [59,60]. In our study, LPS from *A. muciniphila* was found to exert a much lower effect on DC differentiation and functions compared to *E. coli*- and *S. enterica*-derived endotoxins at comparable doses. However, it failed to induce LPS tolerance and to counteract or attenuate the subsequent cell response to *E. coli* LPS, in keeping with recent data reported by Young and colleagues in mouse microglia [47]. This observation would suggest the involvement of cellular receptors other than TLR4, as previously highlighted for other bacterial strains [61]. However, the finding that at higher concentrations *A. muciniphila* LPS can elicit similar effects on DCs as *E. coli* LPS and can induce tolerance toward *E. coli* LPS would claim of a role of TLR4 in its recognition. In line with this hypothesis, Garcia-Vello and colleagues recently demonstrated that *A. muciniphila* LPS exhibits an unprecedented chemical structure being devoid of the O-antigen and carrying a unique mono-phosphorylated lipid A consisting of a balanced mixture of tetra-, penta- and hexa-acylated forms [62]. This unique structure of *A. muciniphila* LPS preferentially activated the TLR2 pathway in human TLR-transfected Hek293 cells and exhibited a low proinflammatory potential in animal studies [62]. Our findings further confirm that different and complex interactions, with different affinities, can occur among TLR4 and LPS from different commensal and pathogenic bacteria, and highlight the need to improve our understanding of the structures and mechanisms of action of endotoxins.

From a functional point of view, the failure of *A. muciniphila* LPS in inducing tolerance toward other endotoxins and cross-tolerance toward viral molecular patterns is consistent with the hypothesis that circulating endotoxins in eubiosis conditions do not affect the ability of immune cells to promptly respond to invading microorganisms. In contrast, it is well known that dysbiosis and associated endotoxemia strongly complicate the pathogenesis of viral diseases [63,64].

Immunosuppression represents a key feature in conditions of chronic inflammation. DCs play a crucial role in this process by inducing T cell anergy and releasing anti-inflammatory cytokines. Host systemic signals and foreign and endogenous factors within the tissue microenvironment can influence DC differentiation and switch their functions toward immunity or tolerance [32]. In particular, in the TME, DCs cannot exert their proper function due to the state of immune tolerance enforced by the tumor milieu that suppresses antitumor T cell response [65]. Increased PD-L1 expression in both tumor cells and infiltrating myeloid immune cells has been documented in many cancers as a promoter of tumorigenesis and/or the tumor immune escape mechanism, and strategies aimed at blocking PD-1 and CTLA-4 inhibitory signaling are being successfully used in several cancer types [66]. However, the factors and mechanisms forcing inhibitory molecule expression have been only partially identified. In this regard, selective LPS blocking inside CRC tissue was shown to attenuate metastatic cancer progression and boost PD-L1-targeting immunotherapy [17,18]. Here, we show that monocyte precursor priming with low-dose pathogenic-bacteria-derived endotoxins, which can be found in the circulation or intestinal mucosa in dysbiosis condition, results in an increased proportion of PD-L1-expressing DCs that could contribute to the immunosuppressive microenvironment. Moreover, the concomitant expression of PD-L1 and CD80/B7-1 could further regulate the inhibitory activity and the response to immune checkpoint inhibitors, although contrasting mechanisms have been proposed regarding this issue [67,68,69]. Although sex dimorphism in LPS-induced soluble PD-L1 release has been described in endothelial cells [49], no sex specificities were identified in this study, neither in the baseline PD-L1 expression nor in its induction by LPS conditioning. However, differences in in vivo PD-L1 induction cannot be excluded, since lower levels of circulating LPSs have been described in females compared to males [70].

Since PD-L1 is described as a key negative regulator of DC function and inducer of tolerance, we further analyzed its regulation, investigating STAT3 activation in LPS-stimulated DC cultures. STAT3 has been reported to be induced by both IL-6 and IL-10 and to regulate the expression of PD-L1 in DCs [50]. Nevertheless, we observed an almost constitutive STAT3 activation in all conditions, suggesting that different kinetics or additional mechanisms might be involved in LPS-induced PD-L1 regulation. In this regard, several mechanisms of temporal regulation of the activity of this transcription factor have been previously reported [71].

We also demonstrate for the first time that LPS-conditioned DCs acquire the ability to suppress IFN-γ production in neighboring γδ T lymphocytes. As cytotoxic cells and a major innate source of IFN-γ, γδ T cells are considered relevant players in natural host defense against cancer and potent MHC-unrestricted effectors of antitumor response [35]. Thus, it can be speculated that, by affecting these cells, endotoxins might contribute to tumor immune escape. Furthermore, we report that, in contrast to what was recently reported in a mouse model of experimental LPS-induced inflammation [72], LPS conditioning of DCs is not sufficient to drive human γδ T lymphocytes toward IL-17 production. We have previously shown that IFN-γ production by aminobiphosphonate-activated γδ T lymphocytes is strongly inhibited by IL-10 [73]. Although the low number of circulating γδ T cells recovered did not allow us to explore this issue, the high IL-10 production induced by *E. coli* and *S. enterica* LPS priming could contribute to IFN-γ suppression. Likewise, it can be hypothesized that PD-L1 overexpression may, in turn, contribute to IFN-γ reduction in γδ T lymphocytes. In this regard, suppression of the PD-1/PD-L1 axis was reported to increase IFN-γ production by ZOL-activated γδ T cells, without affecting cell activation and expansion [74].

As sentinels in tumor surveillance and potent effectors of antitumor response, γδ T cells have become attractive candidates for alternative cancer immunotherapy strategies, and amino-bisphosphonate drugs, such as zoledronate, are being exploited to trigger their ex vivo or in vivo activation, expansion, and IFN-γ production [38]. In this context, we may envisage that the expression of inhibitory molecules and immunosuppressive cytokines in DCs following early precursor encounters with pathogenic-bacteria-derived endotoxins, either in the blood or in the TME, could not only suppress the anticancer activity of γδ T lymphocytes but also act as a brake on γδ T cell-based immunotherapy. These data open a key issue in the context of γδ T cell targeted immunotherapy, suggesting the need for combined strategies aimed at boosting Vδ2 T cells while circumventing suppressive mechanisms.

## Data Availability

Data are contained within the article and Supplementary Materials.

## Supplementary Materials

The following supporting information can be downloaded at: https://https://www.mdpi.com/article/10.3390/biom14121571/s1. Figure S1: Morphologic features and viability of DC cultures generated from human monocytes; Figure S2: Synergic and antagonistic effects of different LPS on surface PD-L1 and CD80 expression. Figure S3: Sex differences in surface PD-L1 expression on human DC cultures conditioned with LPS; Figure S4: Phenotypic and antigen processing analyses of LPS-conditioned DCs; Figure S5: Effect of monocyte priming with LPS from the different bacterial strains on DC maturation induced by E. coli LPS; Figure S6: Effect of monocyte priming with LPS from different bacterial strains on DC maturation induced by R848; Figure S7: Effect of monocyte precursor priming with LPS from the different bacterial strains on DC-mediated TCRVδ2 T cell expansion.
