# Supplementary material for: Akkermansia muciniphila- and Pathogenic Bacteria-Derived Endotoxins Differently Regulate Human Dendritic Cell Generation and γδ T Lymphocyte Activation
Source: Biomolecules. 2024 Dec 9;14(12):1571. doi: 10.3390/biom14121571 (PMC11673428; doi:10.3390/biom14121571)
Supplement: Supplementary file 1 [file biomolecules-14-01571-s001.zip › biomolecules-3305909-supplementary.pdf]

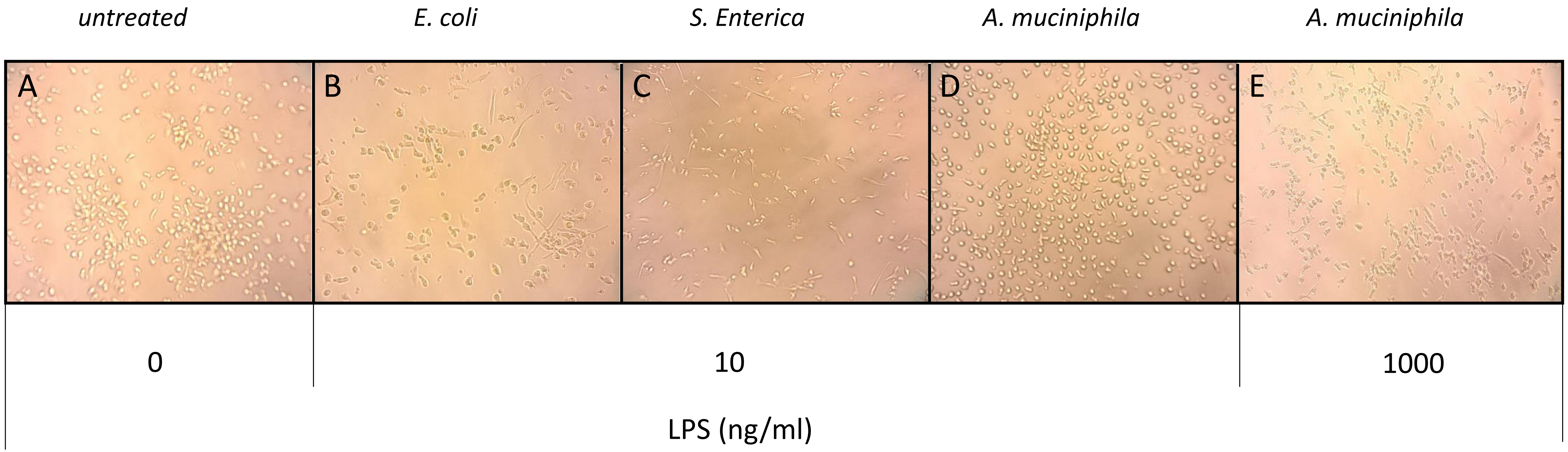

LPS	ng/ml	Cell Viability (%)
-	-	97 ± 4,6
E. coli	10	73 ± 1,7
S. enterica	10	67 ± 10,6
A. muciniphila	10	92 ± 4,4
	1000	77 ± 6,7

Figure S1. Morphologic features and viability of DC cultures. Cell morphology, observed under the light microscope, of DC generated from human monocytes left unprimed (A) or primed with 10 ng/ml of E. coli (B), S. enterica (C), A. muciniphila (D) and 1000 ng/ml of A. muciniphila (E) derived LPS is shown in the top panel. The percentages of live cells, as assessed by Trypan blue dye staining, in unstimulated and LPS conditioned DC cultures is shown in the bottom panel. Results are expressed as mean ± SD of four independent experiments.

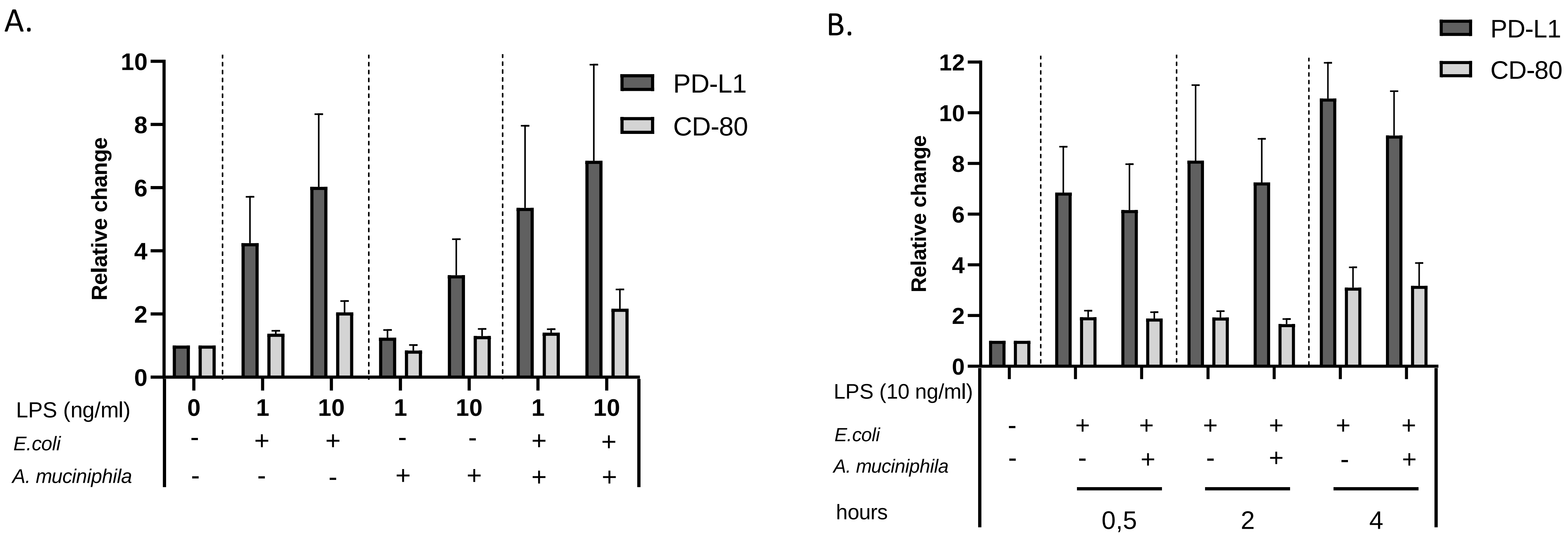

Figure S2. Synergic and antagonistic effects of A. muciniphila and E. coli LPS on surface PD-L1 and CD80 expression. Human peripheral blood monocytes were simultaneously exposed to A. muciniphila and E. coli LPS (A), or treated with A. muciniphila LPS for 30 minutes, 2 or 4 hours before priming with E. coli LPS or treated with E. coli LPS alone at the indicated time points (B), respectively. Cells were analyzed at day 5 of culture by flow cytometry for the expression of surface PD-L1 and CD80. The relative change of the percentages of PD-L1 and CD80 positive cells in LPS-treated versus untreated cultures is reported in (A) and (B). Mean values ± SE from 3 to 4 independent donors are shown.

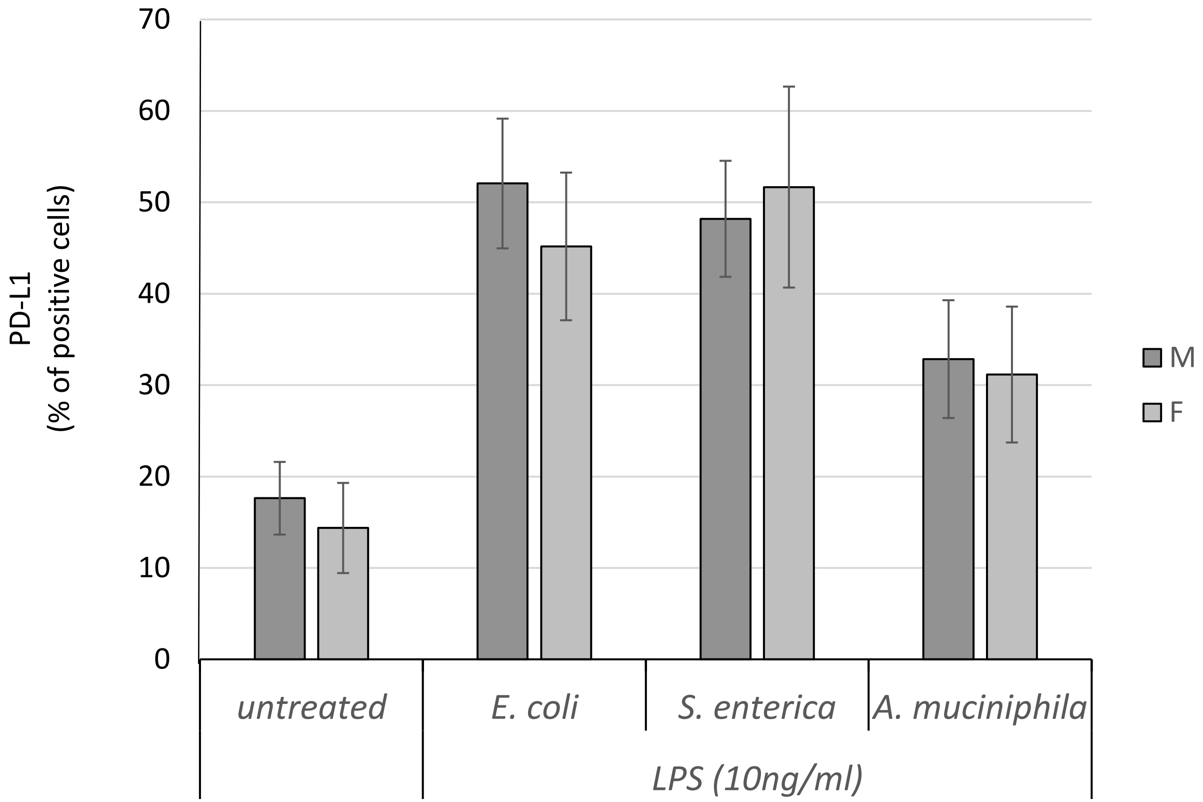

Figure S3. Sex differences in surface PD-L1 expression on human DC cultures conditioned with LPS. Human peripheral blood monocytes were induced to differentiate toward DC in standard medium in the absence or presence of 10 ng/ml of LPS from E. coli, S. enterica and A. muciniphila. Cells were analyzed at day 5 of culture by flow cytometry for the expression of surface PD-L1. The percentages of PD-L1 positive cells in LPS-primed versus unprimed cultures is reported. Mean values ± SE from 13 male and 8 female subjects are shown.

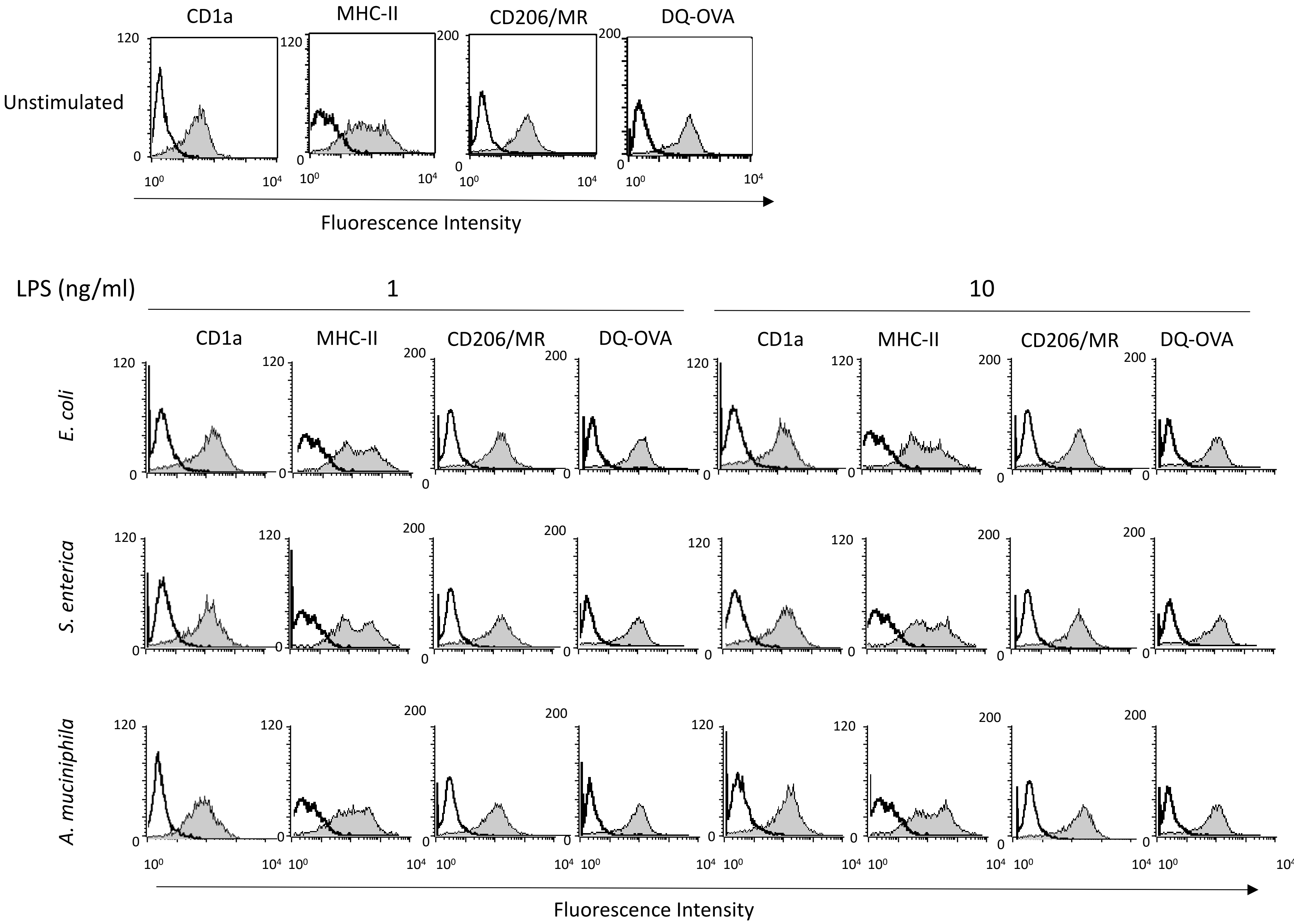

CD1a
MHC-II
CD206/MR
DQ-OVA
Unstimulated
Fluorescence Intensity
LPS (ng/ml)
1
10
E. coli
S. enterica
A. muciniphila
120
200
0
10^0
10^4

Figure S4. Phenotypic and antigen processing analyses of LPS conditioned DC. DC cultures were generated in the absence or presence of the indicated doses of LPS from E. coli, S. enterica and A. muciniphila, and analyzed at day 5 for the surface expression of CD1a, MHC class II and CD206/MR or for ovalbumin processing, by flow cytometry. Empty histograms represent the background staining of cells incubated with isotype-matched control mAbs. For antigen processing analysis cells were pulsed with DQ Ovalbumin (DQ-OVA) conjugate for 15 min at 37°C, washed, and incubated further for 30 min at 37°C to allow proteolytic degradation. Ovalbumin processing was analyzed by measuring the increase of cell-associated fluorescence. Empty histograms represent the background staining of cells pulsed with DQ-OVA and kept at 0°C. Results from one representative donor out of four are shown.

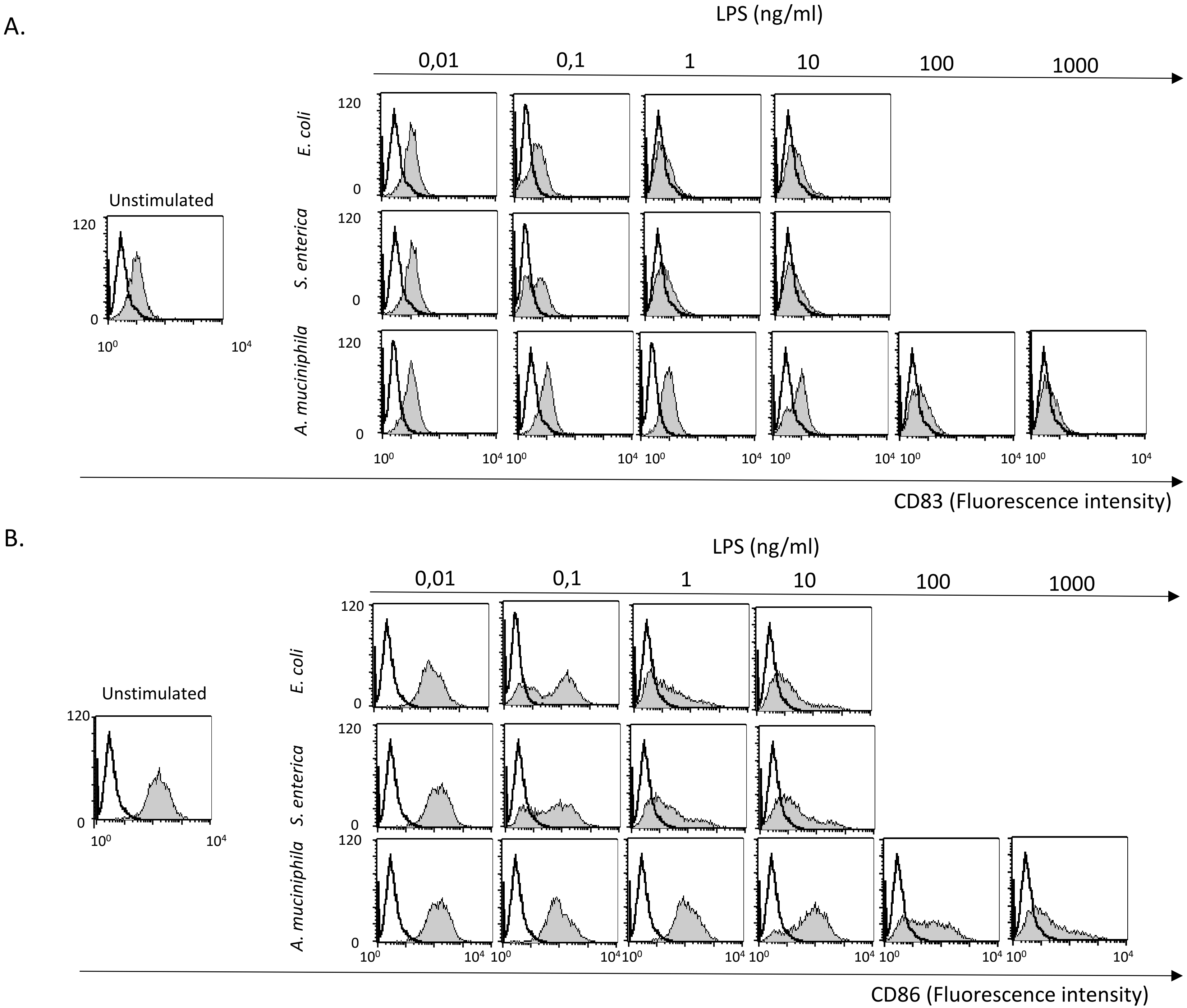
A.
LPS (ng/ml)
0,01
0,1
1
10
100
1000
Unstimulated
E. coli
S. enterica
A. muciniphila
120
0
10^0
10^4
CD83 (Fluorescence intensity)
B.
LPS (ng/ml)
0,01
0,1
1
10
100
1000
Unstimulated
E. coli
S. enterica
A. muciniphila
120
0
10^0
10^4
CD86 (Fluorescence intensity)

Figure S5. Effect of monocyte priming with LPS from the different bacterial strains on DC maturation induced by E. coli LPS. DC were differentiated from monocytes exposed or not to increasing concentrations of the different LPS and then left untreated or stimulated with E. coli LPS during the last 24 hours of culture to induce maturation. The expression of the surface activation markers CD83 (A) and CD86 (B) was analyzed by flow cytometry. One representative experiment out of ten performed is shown. Empty histograms represent the background staining of cells incubated with isotype-matched control mAbs.

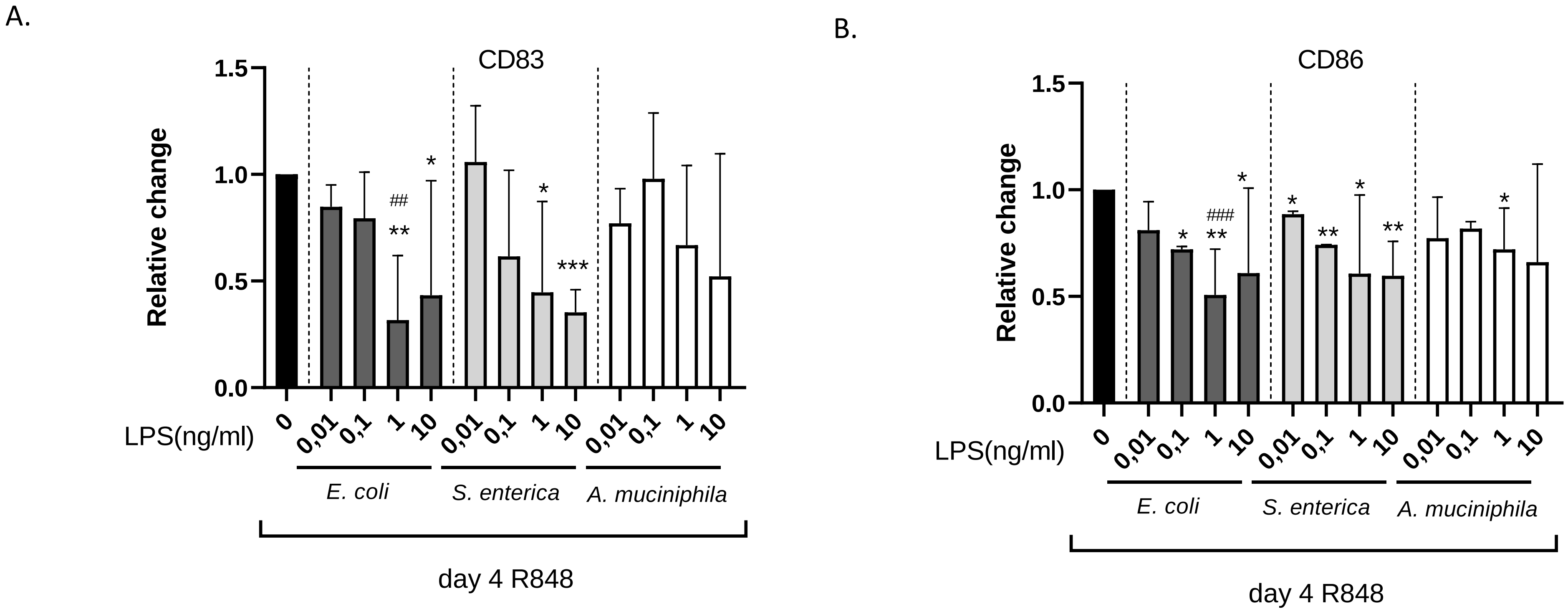

Figure S6: Effect of monocyte priming with LPS from different bacterial strains on DC maturation induced by R848. DC were differentiated from monocytes exposed or not to increasing concentrations of the different LPS and then left untreated or stimulated with the TLR8 ligand R848 during the last 24 hours of culture to induce maturation. (A-B) The expression of the surface activation markers CD83 and CD86 was analyzed by flow cytometry. Histograms indicate the relative changes of the percentages of positive cells in LPS-primed versus unprimed cultures. Results are shown as mean values ± SE from six independent donors. Statistical significance was indicated versus untreated control (*) or versus comparable concentrations of A. muciniphila LPS (#). * 0.01<p ≤ 0.05; **/## 0.001<p ≤ 0.01; ***/### p ≤ 0.001.

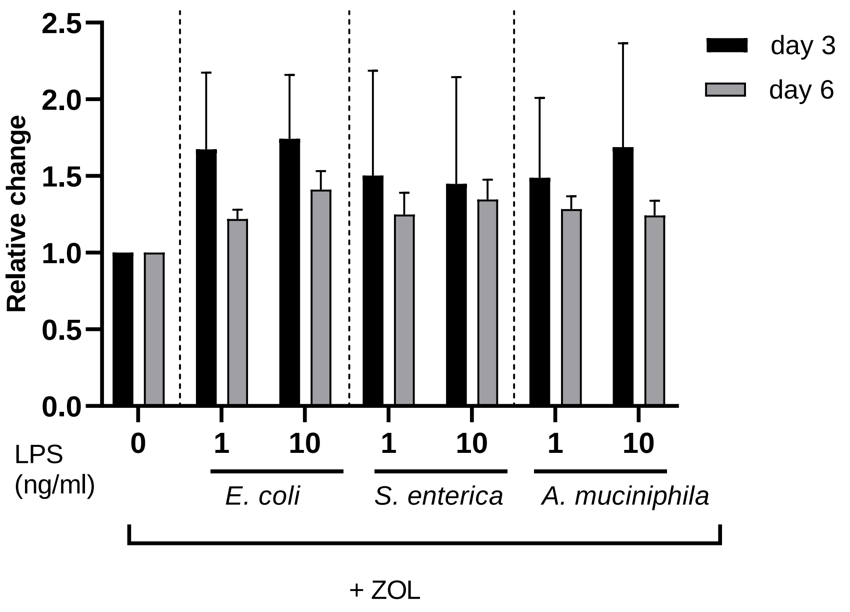

Figure S7: Effect of monocyte precursor priming with LPS from the different bacterial strains on DC-mediated TCRVδ2 T cell expansion. DC generated in the absence or presence of LPS from the indicated bacterial strains were cocultured with autologous PBL (1:1 ratio) and left unstimulated or stimulated with ZOL. Expansion of TCRVδ2 T cells within total PBL was analyzed after 3 and 6 days by flow cytometry gating on the lymphocyte population. The relative change of the percentages of TCRVδ2 positive cells in LPS primed versus unprimed ZOL stimulated cocultures is reported. Mean values ± SE from 4 independent donors are shown.
